# Distinct microRNA and protein profiles of extracellular vesicles secreted from myotubes from morbidly obese donors with type 2 diabetes in response to electrical pulse stimulation

**DOI:** 10.3389/fphys.2023.1143966

**Published:** 2023-03-30

**Authors:** Vigdis Aas, Reidun Øvstebø, Berit Sletbakk Brusletto, Trude Aspelin, Anne-Marie Siebke Trøseid, Saba Qureshi, Desima Shitandi Otundo Eid, Ole Kristoffer Olstad, Tuula A. Nyman, Kari Bente Foss Haug

**Affiliations:** ^1^ Department of Life Sciences and Health, Oslo Metropolitan University (OsloMet), Oslo, Norway; ^2^ Department of Medical Biochemistry, Oslo University Hospital, Ullevål, Oslo, Norway; ^3^ Department of Immunology, University of Oslo, and Oslo University Hospital, Rikshospitalet, Oslo, Norway

**Keywords:** human myotubes, extracellular vescicles, microRNA, electrical pulse stimulation (EPS), proteomic, transcriptomic, morbid obesity, type 2 diabetes (T2D)

## Abstract

Lifestyle disorders like obesity, type 2 diabetes (T2D), and cardiovascular diseases can be prevented and treated by regular physical activity. During exercise, skeletal muscles release signaling factors that communicate with other organs and mediate beneficial effects of exercise. These factors include myokines, metabolites, and extracellular vesicles (EVs). In the present study, we have examined how electrical pulse stimulation (EPS) of myotubes, a model of exercise, affects the cargo of released EVs. Chronic low frequency EPS was applied for 24 h to human myotubes isolated and differentiated from biopsy samples from six morbidly obese females with T2D, and EVs, both exosomes and microvesicles (MV), were isolated from cell media 24 h thereafter. Size and concentration of EV subtypes were characterized by nanoparticle tracking analysis, surface markers were examined by flow cytometry and Western blotting, and morphology was confirmed by transmission electron microscopy. Protein content was assessed by high-resolution proteomic analysis (LC-MS/MS), non-coding RNA was quantified by Affymetrix microarray, and selected microRNAs (miRs) validated by real time RT-qPCR. The size and concentration of exosomes and MV were unaffected by EPS. Of the 400 miRs identified in the EVs, EPS significantly changed the level of 15 exosome miRs, of which miR-1233-5p showed the highest fold change. The miR pattern of MV was unaffected by EPS. Totally, about 1000 proteins were identified in exosomes and 2000 in MV. EPS changed the content of 73 proteins in exosomes, 97 in MVs, and of these four were changed in both exosomes and MV (GANAB, HSPA9, CNDP2, and ATP5B). By matching the EPS-changed miRs and proteins in exosomes, 31 targets were identified, and among these several promising signaling factors. Of particular interest were CNDP2, an enzyme that generates the appetite regulatory metabolite Lac-Phe, and miR-4433b-3p, which targets CNDP2. Several of the regulated miRs, such as miR-92b-5p, miR-320b, and miR-1233-5p might also mediate interesting signaling functions. In conclusion, we have used a combined transcriptome-proteome approach to describe how EPS affected the cargo of EVs derived from myotubes from morbidly obese patients with T2D, and revealed several new factors, both miRs and proteins, that might act as exercise factors.

## Introduction

Lifestyle disorders like obesity, type 2 diabetes (T2D), and cardiovascular diseases are continuously growing global challenges. Lifestyle interventions including regular physical activity is first line treatment, particularly for T2D. Regular physical activity is associated with reduced morbidity and mortality risk, and a robust inverse association between exercise level and all-cause mortality has been shown (Arem et al., 2015). The role of physical activity in preventive and therapeutic medicine is emerging, and exercise prescription protocols for various conditions are being developed (Luan et al., 2019). The most recent consensus report from The American Diabetes Association (ADA) and the European Association for the Study of Diabetes (EASD) on management of hyperglycemia in type 2 diabetes recommend regular, aerobic physical activity of moderate to vigorous intensity >150 min/week (Davies et al., 2022). Physical exercise improves skeletal muscle strength and performance, and promotes bone and skeletal muscle anabolism, in addition to having a huge impact on whole body healthiness. Beneficial effects on cardiovascular system have undoubtedly been observed (Fiuza-Luces et al., 2018; Adams and Linke, 2019), metabolic regulation and weight control are improved (Zhao et al., 2021), the risk of certain cancers is reduced (McTiernan et al., 2019), and even cognitive function and mental health seem to be improved by physical exercise (Pedersen, 2019).

The comprehensive effects of physical exercise imply an effect of skeletal muscle activity beyond the contracting skeletal muscle and suggest that skeletal muscles in some way communicate with other distant organs. How this occurs is not completely understood. It is known that skeletal muscle can act as an active secretory organ, and that contraction can change the skeletal muscle secretome (Giudice and Taylor, 2017). An array of myokines and signaling metabolites are released from contracting skeletal muscles (Hartwig et al., 2014). Interleukin-6 (IL-6) was the first exercise factor to be identified (Pedersen et al., 2003), and later several myokines have been shown to be both up- (Covington et al., 2016) and downregulated (Hjorth et al., 2016) by exercise stimulation. In addition to myokines, many metabolites like lactate and ketone bodies are markedly increased in the circulation after intense exercise (Schranner et al., 2020). Recently, the metabolite N-lactoyl-phenylalanine (Lac-Phe) was shown to be induced by exercise and play an important role in food intake and weight control (Li et al., 2022a). Adding to the complexity of the total secretome of skeletal muscles, extracellular vesicles (EVs) are emerging as important exercise factors (Trovato et al., 2019; Estebanez et al., 2021).

Extracellular vesicles (EVs) are a heterogeneous population of nano-sized vesicles produced by almost any type of cell and can be found in all body fluids. These vesicles vary in size, content, and mechanism of production. Although there is not a clear cut between EV subgroups, one way of grouping them is by size and biosynthesis. Exosomes are the smallest (30–150 nm) formed by fusion of multivesicular endosomes with the cell membrane, whereas microvesicles (100–1000 nm) are formed by outward budding of the cell membrane, and the largest vesicles, apoptotic bodies, are formed during cell apoptosis. They are all spherical, lipid bilayer vesicles that contain bioactive molecules, such as lipids, proteins, and nucleic acids, and they participate in cellular communication and regulation. It has been shown that EV number and/or content change with maternal cell status, thus reflecting the health condition of the cells, representing a snapshot of their cell of origin (Kalluri and LeBleu, 2020). In metabolic diseases, such as obesity and T2D, circulating EVs might function both as biomarkers of the disease, as well as contributors to the development of insulin resistance and diabetic complications (Akbar et al., 2019; Castano et al., 2019). The number of circulating EVs has been found to correlate positively with systolic and diastolic blood pressure, BMI, and HOMA-IR (insulin resistance index) (Kobayashi et al., 2018). Evidence suggests that EVs from metabolically active tissues, such as adipose tissue, can transmit insulin resistance throughout the body, and thereby contribute to the pathology of T2D (Li et al., 2022b). Particularly, is the microRNA (miR) content of EVs studied, where let-7, miR-223, miR-29, and miR-107 are known to regulate metabolic disorders, including diabetes (Chang and Wang, 2019). However, EVs might also have the capacity to transfer beneficial signals through intercellular crosstalk between multiple tissues and organs. Based on the origin and physiological state of the parental cells, the EVs may contain components with pronounced regulatory capacity ready for uptake and information exchange in target cells. The number of circulatory EVs increases in an intensity-dependent manner in response to endurance exercise, and it is proposed that the content of some subtypes of EVs may contribute significantly to the systemic benefits of exercise, although there is limited evidence so far (Safdar et al., 2016).

Skeletal muscle cells can release EVs (Guescini et al., 2010; Le Bihan et al., 2012; Romancino et al., 2013; Forterre et al., 2014). EVs have been isolated and characterized from murine skeletal muscle cells (C2C12 cells) (Guescini et al., 2010) and primary human myoblasts and myotubes (Le Bihan et al., 2012). Two types of extracellular vesicles that differed in size, and that contained very distinct proteins and RNA cargo, were isolated from human skeletal muscle cells (Le Bihan et al., 2012). It was further shown that these vesicles could fuse with adjacent skeletal muscle cells, deliver their cargo, and probably affect the myogenesis in a paracrine manner (Le Bihan et al., 2012). Certain pharmacological treatments of myotubes with dexamethasone (Hudson et al., 2014) or the pro-inflammatory agents TNF-α and IFN-γ (Kim et al., 2018) have shown that the EV content can change quickly and reflect the cell condition. *In vivo*, it has been shown that acute physical exercise significantly changed the quantity of over 300 proteins in plasma EVs, confirming the dynamics of EVs (Whitham et al., 2018). In this latter study, they additionally revealed that exercise evoked EVs could transfer their protein content into the liver, implying a role in tissue crosstalk during exercise. Also, the level of miRs in plasma EVs increased by exercise in a group of sedentary men (Doncheva et al., 2022). However, the origin of plasma EV is manifold, and the contribution of skeletal muscle EVs is unknown, although, it has been suggested that about 5% of plasma EVs are derived from skeletal muscles (Estrada et al., 2022). The aim of the present study was to examine how skeletal muscle activity affects size, number, and content of EVs derived from primary myotubes from morbidly obese female patients with T2D. An *in vitro* model of physical activity, electrical pulse stimulation (EPS) (Nikolic et al., 2012) of cultured myotubes, was used to study the protein and RNA patterns of exosomes and MV isolated from the cell media 24 h after EPS. EPS-treatment of myotubes in culture is a model of exercise or physical activity, which has been shown to induce many of the well-known metabolic effects of exercise (Nikolic et al., 2012; Feng et al., 2015). Recently, we also showed that EPS-treatment of human myotubes led to secretion of many proteins, including new and formerly known myokines (Mengeste et al., 2022). Even though EPS cannot fully replace exercise (Nikolic et al., 2017), we believe this reductionistic approach is useful for exclusive examination of contractile activity on skeletal muscle derived EVs from obese patients with T2D.

## Methods

### Materials

Corning^®^ CellBIND^®^ tissue culture plates (6 well) were from Corning (Schiphol-Rijk, the Netherlands). DMEM-Glutamax™ low glucose with sodium pyruvate, DPBS (without Mg^2+^ and Ca^2+^), foetal bovine serum (FBS), Trypsin-EDTA, gentamicin (50 mg/mL), and amphotericin B were from Thermo Fisher Scientific (Waltham, MA, US). Ultroser G was from Pall (Cergy-Saint-Christophe, France) and insulin (Actrapid^®^ Penfill^®^ 100, IE/mL) from Novo Nordisk (Bagsvaerd, Denmark). Trypan blue 0.4% solution, extracellular matrix gel (ECM), and DMSO were from Sigma-Aldrich (St. Louis, MO, US). RNeasy Mini Kit was from QIAGEN (Venlo, the Netherlands). Protein Assay Dye Reagent Concentrate was from Bio-Rad (Copenhagen, Denmark).

### Ethics approval and consent to participate

The study was approved by the Institutional Review Board and Regional Committee for Medical and Health Research Ethics of South-East Norway REK (S-09078d, 2009/166), and the biopsies were obtained after informed written consent. The study adhered to the Declaration of Helsinki.

### Culturing of human myotubes

Cultures of multinucleated human myotubes were established by activation and proliferation of satellite cells isolated from muscle biopsy samples from *m. Obliquus internus abdominis.* The biopsies were taken from six donors during gastric bypass surgery. All donors were female, average age 49 (±12) years, BMI 45 (±5) kg/m^2^, and all were diagnosed with T2D and treated with peroral antidiabetics (two on monotherapy with metformin, one on metformin and glimepiride and two on unknown peroral medication) or insulin (1 donor). Average HbA_1c_ was 6.7 (±1.2) %, and fasting plasma glucose was 6.8 (±0.9) mmol/L in blood samples collected the day before surgery.

Satellite cells were isolated from biopsy samples by enzymatic digestion (Yasin et al., 1977). Briefly, the muscle biopsies were cut into small pieces, rinsed for adipose tissue, and trypsinized 3 times. The supernatants were collected, and the cells seeded in SkGM™-2 (Skeletal Muscle Cell Growth Medium-2 BulletKit (Lonza, Basel, Switzerland). The cells were split twice before storage in liquid nitrogen, and at each splitting fibroblasts were removed by a pre-plating step (Gaster et al., 2001). For proliferation of myoblasts, DMEM-Glutamax™ (5.5 mM glucose) medium supplemented with FBS (2%) and Ultroser G (2%) was used (Gaster et al., 2001). At approximately 80% confluence, the culture medium was changed to DMEM-Glutamax™ (5.5 mM glucose) supplemented with FBS (2%) and insulin (25 pM) to initiate differentiation into multinucleated myotubes. Both cell media contained gentamicin and amphotericin B. The cells were grown in 6-well plates pre-coated with 1.4% extracellular matrix gel (ECM, SigmaAldrich). The cells were allowed to differentiate for 7 days. During the culturing process muscle cells were incubated in a humidified CO_2_ (5%) atmosphere at 37°C, and medium was changed every second to third day.

### Electrical pulse stimulation of myotubes

Human myotubes grown in 6-well EMC coated Costar plates (Corning^®^) were stimulated *via* carbon electrodes (C-dish, Ionoptics, Ireland) by applying continuous, low-frequency electrical pulse stimulation (EPS) (single, bipolar pulses of 2 m, 30 V, 1 Hz) for 24 h from day 6–7 of the differentiation period, as described previously (Nikolic et al., 2012). Electrical pulses were generated by a self-designed pulse generator (the Electronics Lab, Institute of Chemistry, University of Oslo, Norway), and the effect was investigated by measuring IL-6 concentration in the supernatants before and after EPS. IL-6 was measured spectrophotometrically by sandwich ELISA according to the producer’s protocol (Human IL-6 ELISA kit, SigmaAldrich), and the concentration calculated from a standard curve.

### Exosome and microvesicle isolation

After 24 h of EPS, cell medium was changed to serum free DMEM-Glutamax™ (5.5 mM glucose) with 25 pM insulin, gentamicin, and amphotericin B, 1 mL/well for collecting EVs. The cells were incubated for another 24 h for synthesis and secretion of EVs and then harvested. EV-containing media were collected, and both exosomes and microvesicles (MV) were isolated. Briefly, media from parallel 6-well plates were combined, remnant cells and cell debris were removed by centrifugation at 4500 *g* for 5 min, and the final cell free supernatants were stored at −80 C.

Supernatants were thawed at 37°C, and MV were isolated by centrifugation at 17,000 *g* for 30 min using a fixed-angle Sorvall SS-34 rotor (Kendro Laboratory Products, Newtown, CT, United States). The remaining supernatant was transferred to a new vial for exosome isolation. MV pellets were thoroughly dissolved in the residual supernatant (2 mL) and concentrated using Amicon Ultra-4 100 kDa Centrifugal Filter Devices (Merck Millipore, Cork, Ireland). To exclude particles greater than 220 nm in the exosome preparation, the 17,000 g supernatant was gently filtered through a 0.22 μm filter (Merk Millipore) before exosomes were isolated by Centricon-70 Plus 100 kDa Centrifugal Filter Columns (Merk Millipore). Column units were centrifuged at 3500 *g* for 15 min, filters washed with PBS and further centrifuged at 3500 *g* for 10 min. Finally, the filters were turned upside down, centrifuged at 1000 *g* for 2 min, and the exosome concentrates were collected. All centrifugations were performed at room temperature (RT), and all samples were stored in aliquots at −80°C.

### EV characterization

#### Transmission electron microscopy (TEM) analysis

For negative staining, freshly isolated EV samples were stored on ice until fixed by 2% paraformaldehyde (PFA) for 5 min and further attach to formvar/carbon coated grids for 20 min at RT. The grids were then washed with PBS, followed by double distilled water, and stained with 0.4% uranyl acetate/1.8% methyl cellulose for 2 min before excess solution was gently removed by a filter paper and dried. Subsequently, the samples were observed using a FEI CM200 transmission electron microscope at 120 kV, and the images were recorded with a Quemesa CCD digital camera (Olympus Soft Imaging Solutions).

#### Nanoparticle tracking analysis (NTA)

The EVs were analyzed with a nanoparticle tracking analysis instrument (NS500, Malvern, Amesbury, United Kingdom) to determine the vesicle concentration and size distribution. The EV samples were diluted by PBS (0.02 μm-filtered; Whatman Anotop™25, GE Healthcare Life Science, Buckinghamshire, United Kingdom) to be within the recommended concentration range (1.0-9.0×10^8^ particles/mL). Samples were loaded into the NS500 instrument at a constant flow with a syringe pump speed of 20. Triplicates of 60-s videos were captured for each sample. The videos were analyzed by NTA 3.1 software (Malvern). The vesicle quantifications, as previously described, had an intra-assay coefficient of variance (CV) below 7% and inter-assay CV (day-to-day) < 20% (Vestad et al., 2017).

#### Western immunoblotting

To characterize EVs, selected EV (CD9, CD63 and Hsc70/Hsp70) and cell contamination (calnexin) markers (Thery et al., 2018) were assessed by Western immunoblotting. Exosome solution (20 µL) was added RIPA 5x buffer (Thermo Fisher Scientific) and protease inhibitor (cOmplete, Mini, EDTA-free Protease Inhibitor Cocktail 25x, Roche), sonicated for 20 s and lysed on ice for 15 min. Then, LDS Sample Buffer (Invitrogen) was added. Heat shock protein 70 (Hsc70/Hsp70) and calnexin were analyzed under reducing conditions using Bolt Sample Reducing Agent, whereas CD9 and CD63 were measured after addition of PBS. All lysates were heated for 10 min at 70°C. The proteins were separated on Bolt 4%–12% Bis-Tris Plus Gels with Bolt MES SDS Running Buffer (both Invitrogen) and then transferred to 0.2 µm PVDF Blotting Membranes (Invitrogen). The membranes were blocked with 1% casein (Western Blocking Reagent, SigmaAldrich) in tris-buffered saline with 0.1% Tween 20 (TBS-T) for 1 h at RT and subsequently incubated with primary antibodies overnight at 4°C. The membranes were then washed three times (20 min) with TBS-T before incubation with horseradish peroxidase-coupled secondary antibody (Mouse TrueBlot Ultra, Rockland Immunochemicals) for 1 hour at RT. Following triplicate washing with TBS-T, blots were imaged using SuperSignal West Dura Extended Duration Substrate (Thermo Scientific) and Amersham Imager 600 (GE Healthcare). Primary mouse monoclonal antibodies used were anti-CD9 (#10626D, 1:750), anti-CD63 (#10628D, 1:500), anti-calnexin (#MA5-15389, 1:1000) (all from Invitrogen) and anti-Hsc70/Hsp70 (#ADI-SPA-820, 1:1500) (Enzo). Lysed SW480 cells were used as control.

#### Flow cytometry detection of tetraspanin positive EVs

Immunoaffinity detection of CD9, CD63, and CD81 outer membrane markers on EVs from exosome and MV suspensions were performed using the Exosome Human CD9, CD63, and CD81 Flow Detection Kits (catalog number: 10620D, 10606D, and 10622D, Life Technologies AS, ThermoFisher Scientific, Oslo, Norway). Concisely, EV solutions (30 µL) mixed with assay buffer (70 µL) were incubated overnight with anti-CD9, -CD63, and -CD81 coated Dynabeads (2.7 mm) at 4°C. The bead bound EVs were then washed three times with 0.1 µm filtered PBS containing 0.1% bovine serum albumin (BSA). Subsequently, the bead-suspension was incubated with different RPE-conjugated detection antibodies or isotype control (IgG1-RPE, BD Biosciences, Oslo, Norway) for 45 min at RT protected from light. The bead-containing samples were further washed twice before proceeding with flow cytometry analysis, using a BD Accuri™ C6 Cytometer (BD Biosciences, Oslo, Norway). Median fluorescence intensity (MFI) was reported as a signal to noise (S/N) ratio to isotype control from a total of 3000 singlet events.

#### Isolation of RNA from exosomes and MV

Total RNA from the exosomes and MV suspensions were isolated using miRNeasy Micro kit (Qiagen, Hilden, Germany) following the manufacturer´s protocol. In short, up to 150 µL EV-solution was transferred to an Eppendorf tube containing 700 µL Qiazol, incubated in RT, added chloroform, and shaken vigorously, before the tube was incubated for 3 min at RT and centrifuged at 12,000 *g* for 15 min at 4°C. The upper phase was transferred to a new tube, mixed thoroughly with 1.5 volume 100% ethanol, and filled into a RNeasy Min Elute column before a short centrifugation step at 8000 g. Flow-through was discarded, the column washed with buffers RWT and RPE, centrifuged at 8000 *g* before 80% ethanol was added, and the column centrifuged dry with open lid. Finally, RNA was eluted in 14 µL nuclease free water and stored at −80°C until analyzed.

#### Isolation of total RNA from myotubes

Remaining myotubes were disrupted by adding 150 µL RLT Plus Lysis buffer (Qiagen) to each well of the 6-well culturing plate. Cell lysates were incubated at 4°C for 15 min and shaken, before further storage at −20°C. Total RNA was isolated by combining two procedures; long RNA (>200 bp) was extracted using RNeasy Plus Mini-kit (Qiagen), whereas short RNA (<200 bp) was isolated from the flow-through of the first procedure by the RNeasy MinElute Cleanup kit (Qiagen), essentially following the manufacturer´s protocols.

Lysates were gently thawed, thoroughly vortexed, and transferred to a gDNA Eliminator spin column for centrifugation at 8000 *g* for 15 s in RT. Flow-through was added one volume of 70% ethanol, mixed by pipetting, and immediately loaded on a RNeasy Mini spin column for centrifugation at 8000 *g* for 15 s. Whereas the spin column was stored at 4°C until isolation of large RNA, the flow-through was transferred to a new tube, added 0.65 volume 100% ethanol, and mixed by vortexing. The samples were immediately transferred to a RNeasy MinElute spin column and centrifuged at 8000 *g* for 15 s. The columns were washed twice by RPE buffer, added 80% ethanol, and centrifugation was repeated. The columns were placed in a new tube, centrifuged at 8000 *g* for 1 min with open lid and transferred to new 1.5 mL tubes. Finally, small RNA was eluted from the column by 14 µL RNase-free water and centrifuged for 1 min at 8000 g.

#### Quantification of isolated RNA

Isolated RNA was analyzed by NanoDrop One™, Bioanalyzer (Agilent RNA 6000 Pico Kit), and Qubit^®^ (Qubit™ microRNA assay kit) to quantify RNA concentration and for validation of RNA quality, before stored at −80°C until further downstream analysis.

#### Affymetrix analysis

Flash Tag TM Biotin RNA Labeling Kit and the Affymetrix GeneChip TM miRNA 4.0 (ThermoFischer, Waltham, MA, United States) were used for analysis of RNA isolated from exosomes, MV, and myotubes. This kit is designed for analysis of miRNA from cells (130–100 ng), and a pilot experiment with different amounts of RNA from EVs was performed to ascertain the optimal input of RNA needed to achieve reproducible data to enable the comparison of miR cargo of EVs from unstimulated and EPS-treated cells. Satisfactory results were achieved from 1–10 ng of EV RNA, and 2.5 ng was chosen as RNA input. Exosome, MV, and myotube RNA were subjected to a tailing reaction and biotin-labelling in a hybridization cocktail before hybridization at 48°C for 18 h. The miRNA microarrays were then washed and stained using the Genechip Fluidics Station 450 (Affymetrix, Santa Clara, CA, United States). Signal intensities were detected by a Hewlett Packard (Palo Alto, CA, United States) 30007G gene array scanner, and data from scanned arrays were transferred to the Affymetrix^®^ GeneChip™ Command Console software for normalization of signal intensities and check of in-array quality controls. To evaluate the labelling protocol and array processing, the spike-in control probe sets (Affymetrix, Thermo Fisher) had to achieve signals ≥ 1000. The CEL files were imported into Partek R Genomics SuiteTM software (Partek, St. Louis, MO, United States) for statistical analysis. The Robust Multichip Analysis (RMA) algorithm was applied for generation of signal values and normalization. Each array contained human detection probes for 2578 mature miRNAs, 2025 pre-miRNAs, 1996 other non-coding RNAs (snoRNA, CDBox RNA, H/ACA box RNA, and scaRNA). For signal comparisons of different groups, miRNA profiles were compared using a one-way ANOVA model. The results were expressed as fold change (FC). MiRNAs with FC ≥ ± 1.5 and *p*-value <0.05 were considered significantly regulated. The lists of miRNA data with identifiers and corresponding *p*-values, were imported into Ingenuity Pathway Analysis (IPA) for core analysis to predict enriched pathways. To explore and predict relationships and biological functionality between significant miRNAs and proteins in exosomes, a miRNA-target analysis in IPA was performed.

#### miRNA quantification and validation by RT-qPCR

Isolated RNA from myotube derived exosomes was subjected to TaqMan^®^ Advanced miRNA cDNA Synthesis kit (Thermo Fischer Scientific, CA, United States) for building cDNA libraries of all RNA present, as recommended by the manufacturer´s protocol. The presence of muscle specific myo-microRNAs (myomiRs) were verified by amplification of miR-1-3p, miR-133a-3p and miR-206, by TaqMan^®^ Advanced miRNA Assay kits (Thermo Fischer Scientific, CA, United States) on a ViiA™7 real-time PCR-instrument (Life Technologies, Singapore). For validation of obtained microarray results, miR-23a, miR-92a, and miR-320 were quantified from 2.5 to 25 ng RNA.

#### Proteomics

EVs corresponding to 10 µg of protein from the exosome suspensions (*n* = 12, six donors±EPS) and 6 µg from the MV suspensions (*n* = 6, three donors±EPS) were subjected to proteomic and bioinformatics analysis. The vesicles were lysed with 0.1% ProteaseMax Surfactant, after which the proteins were reduced, alkylated, and digested into peptides with trypsin. The resulting peptide mixture was purified by STAGE-TIP methods using a C18 resin disk (3M Empore) before the samples were analyzed by a nanoLC-MS/MS using nEASY-LC coupled to QExactive (Thermo Electron, Bremen, Germany) with a 60 min LC separation gradient.

The resulting LC-MS/MS files were processed with MaxQuant version 1.6.1.0 for protein identification and label-free quantification (LFQ) using default settings. The searches were done against the human Uniprot Reference Proteome database (January 2020). Perseus software version 1.6.13.0 was used for the statistical analysis of the proteomics data. The reverse entries and known contaminants provided by MaxQuant were removed, LFQ values were log10 transformed, data was filtered to include only entries having a valid value in at least 50% of the samples in at least one group, missing values were imputed from normal distribution, and paired Student’s t-test with *p*-value <0.05 was done. Further bioinformatics analysis for the EXO and MV proteome data was done using FunRich (http://www.funrich.org/) and Ingenuity Pathway Analysis™ (Qiagen).

#### Data processing and statistical analyses

The data are presented as mean ± standard error of the mean (SEM). In cell experiments, the differences between groups were determined with two-sided Student’s t-test (Microsoft^®^ Excel^®^). The correlation between exosome, MV, and myotube miRs was performed with Pearson’s correlation analysis (Microsoft^®^ Excel^®^). *p* values < 0.05 were considered statistically significant.

## Results

### Study workflow and IL-6 concentration in cell media

**FIGURE 1:**
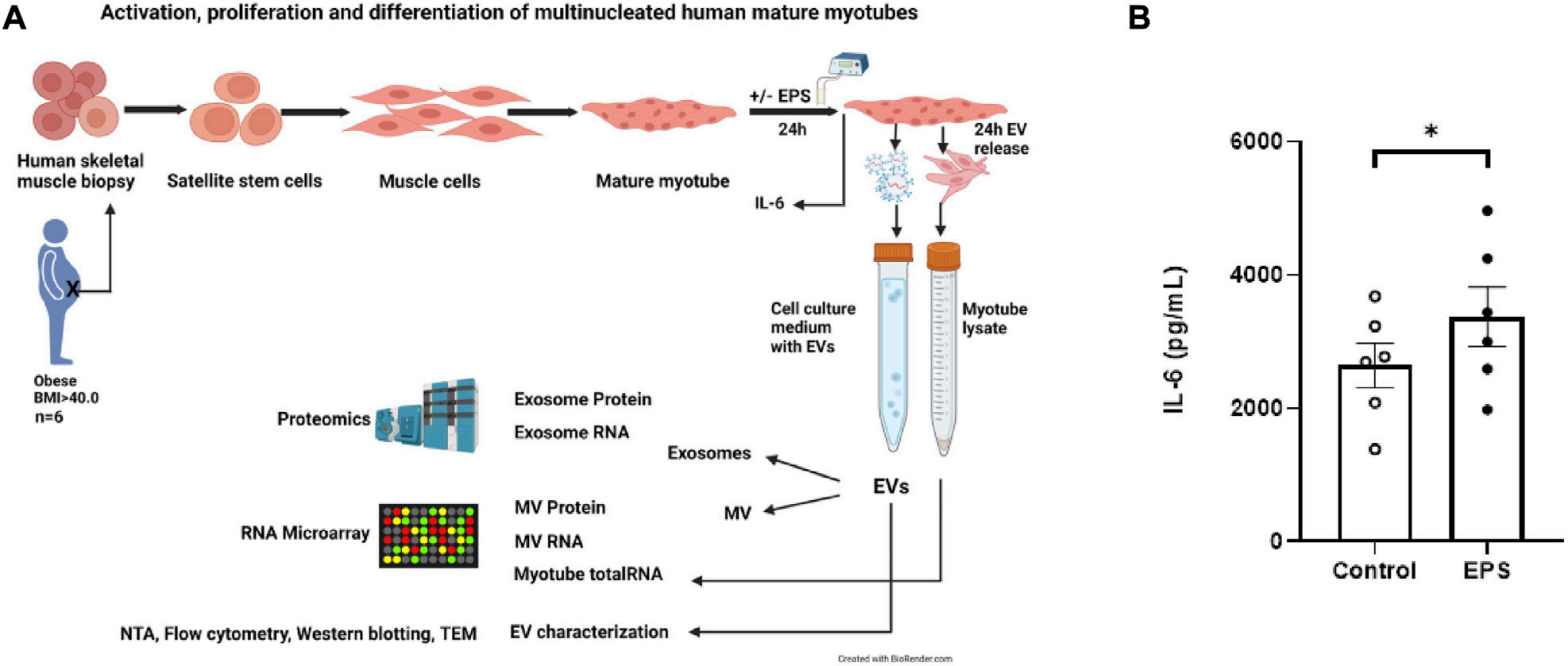
Study workflow and cell medium concentration of interleukin-6 (IL-6). Flow chart of the study from human biopsy sampling, skeletal muscle cell culturing, electrical pulse stimulation (EPS) of myotubes, isolation of extracellular vesicles (EVs) from conditioned medium, separation of exosomes from microvesicles (MV), characterization of EVs by nanoparticle tracking analysis (NTA), flow cytometry, Western blotting, transmission electron microscopy (TEM) to transcriptomic (RNA microarray) and proteomic analysis **(A)**. Cultured human myotubes were exposed to EPS for 24 h, conditioned medium was harvested, and the concentration of IL-6 was detected with a human ELISA kit according to the producer’s protocol (SigmaAldrich). Data are given as mean (±SEM) (*n* = 6). *Significantly different from control (unstimulated cells) *p* < 0.05 **(B)**.

Human skeletal muscle cells were isolated from muscle biopsy samples from morbidly obese females with T2D during bariatric surgery, cultured *in vitro* to multinucleated myotubes, and exposed to EPS. EVs from the cell culture media were isolated, and the EV content of miRNAs and proteins were examined (Figure 1A). Increased release of interleukin-6 (IL-6) by 29% (±9) (Figure 1B) to the cell medium confirmed that the cultured muscle cells had performed contractile activity during EPS (*p* = 0.006).

### Characterization of exosomes and MV secreted from cultured human myotubes

**FIGURE 2:**
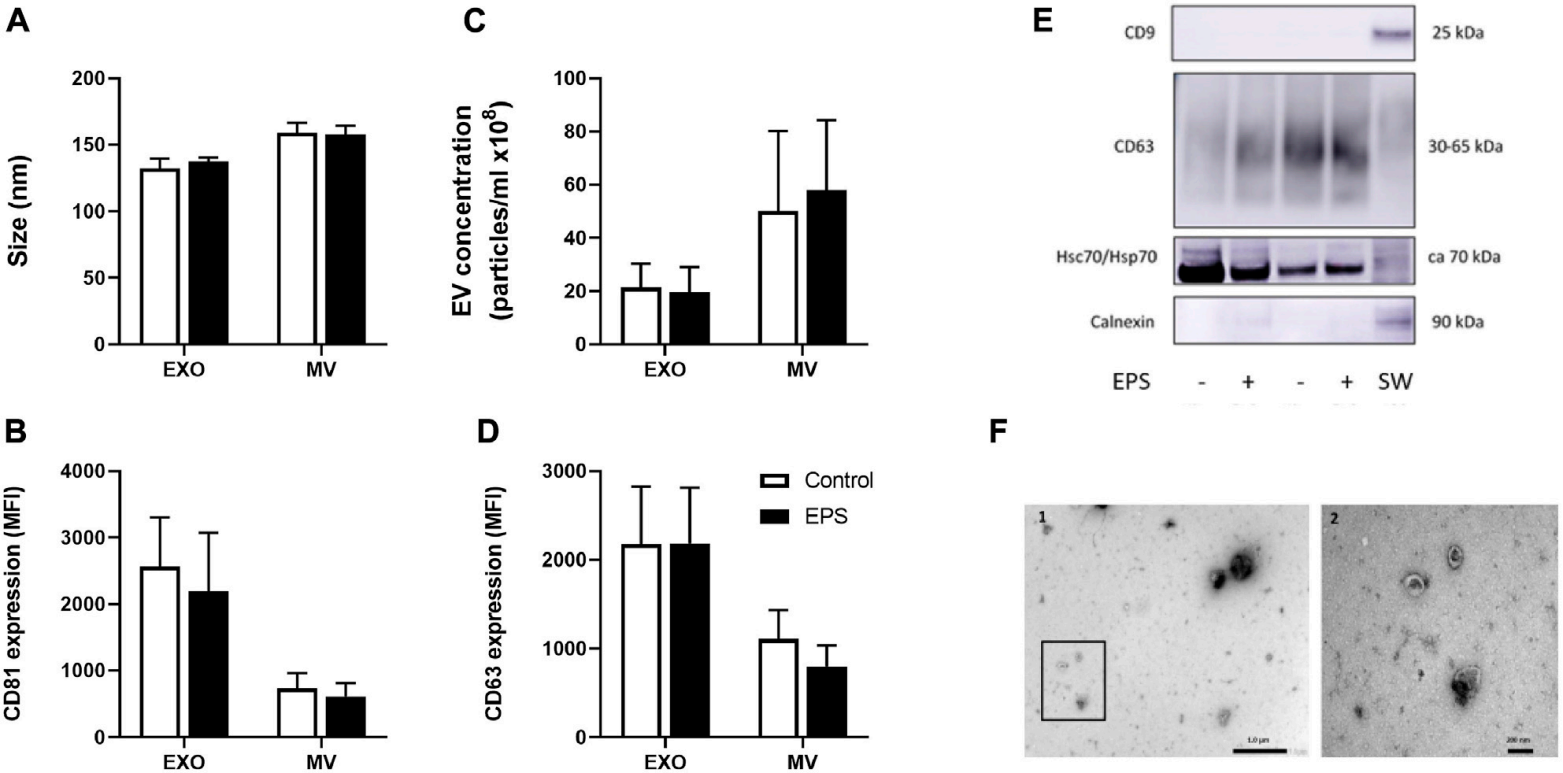
Characterization of myotube derived extracellular vesicles (EVs). Human myotubes were exposed to electrical pulse stimulation (EPS) for 24 h, and cell derived EVs were collected for 24 h thereafter. Size **(A)** and concentration **(C)** of exosomes (EXO) and microvesicles (MV) were measured by nanoparticle tracking analysis (NTA). The presence of EV markers on exosomes and MV captured by anti-CD81-coated magnetic beads were detected with PE-conjugated CD81 **(B)** and CD63 **(D)** antibodies by flow-cytometry (BD Accuri C6 flow cytometer). Presence of CD9, calnexin, and heat shock protein 70 (Hsc/Hsp70) on exosomes were measured by Western blotting **(E)**. Cell lysates of SW480 cells (SW) were used as control. Transmission electron microscopy (TEM) images of skeletal muscle cell derived EVs **(F)**. 1. Freshly isolated EVs from conditioned media from human myotubes, scale bar 1 µm. 2. Close up of framed EVs in picture 1, scale bar 200 nm. Data are presented as mean ± SEM (*n* = 6 in each group). MFI = mean fluorescence intensity.

The mean size of exosomes was 132 nm (±7) and of MV 159 nm (±8) (Figure 2A). Mean concentration of exosomes was 2.2 × 10^9^ particles/mL (±8.7 × 10^8^) and of MV 5.0 × 10^9^ particles/mL (±3.0 × 10^9^) (Figure 2C). Neither size nor concentration were affected by EPS (Figures 2A,C). The presence of the surface markers, the tetraspanins CD9, CD63, and CD81, were assessed by flow cytometry (Figures 2B,D), whereas CD9, CD63, and Hsc70/Hsp70, and the negative marker calnexin were analyzed by Western blotting (Figure 2E). There was no difference between the levels of CD63 and CD81 positive exosomes and MV with or without EPS, but both CD63 and CD81 -signals were elevated on exosomes compared to MV (Figures 2B,D) (*p* = 0.01 and *p* = 0.002, respectively). CD9 was not detected in any of the myotube derived EVs (Figure 2E). Myotube derived EVs were confirmed with the typical cup-shaped EV morphology observed by transmission electron microscopy (TEM) (Figure 2F).

### Microarray analysis of RNA from myotube derived EVs

**FIGURE 3:**
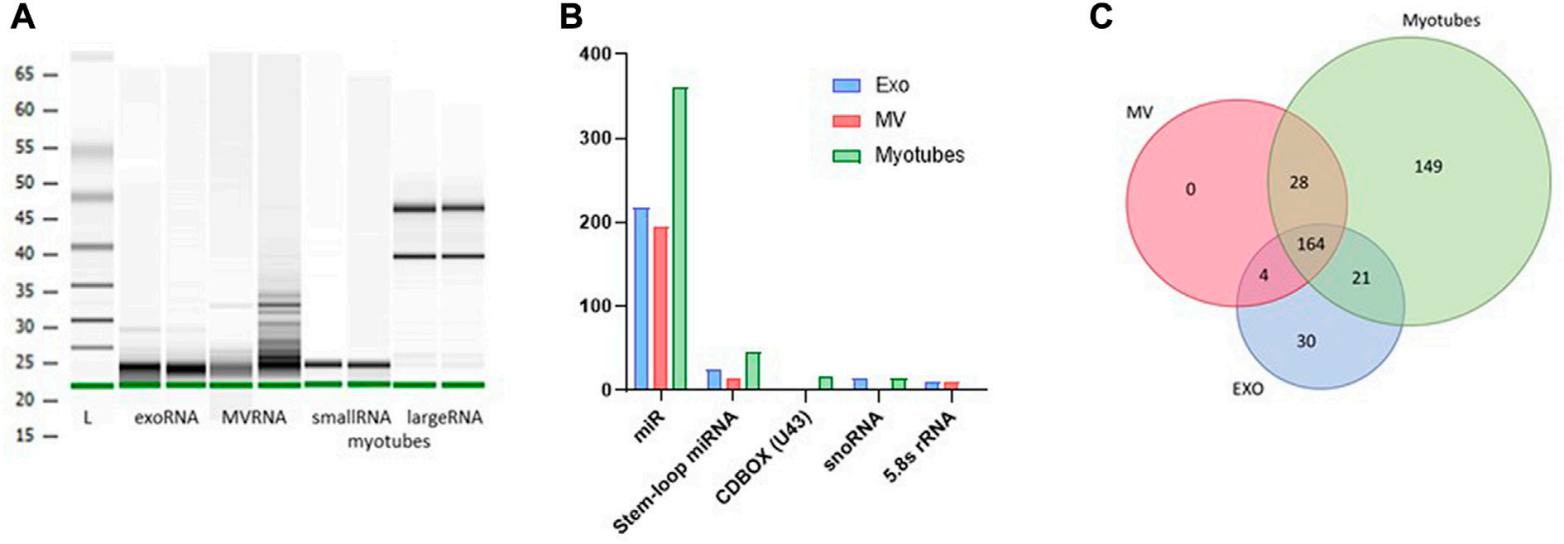
Content of non-coding RNA in exosomes (EXO), microvesicles (MV) and myotubes from unstimulated cells. Quality assessment of isolated RNA from exosomes, MV, and myotubes by Agilent BioAnalyzer with two representative samples of each **(A)**. Small non-coding RNA in exosomes, MV, and myotubes were identified by microarray transcriptomics. Distribution of total non-coding RNA content of EXO, MV, and myotubes **(B)**. The total number of microRNAs (miR) that were unique and common for EXO, MV, and myotubes **(C)**.

All RNA samples utilized for microarray analysis were quality controlled by Agilent Bioanalyzer (Figure 3A). RNA from exosomes and MV showed substantially small sized fragments, and the fraction of small RNAs isolated from myotube total RNA demonstrated a distinct fragment pattern without contamination from large RNAs (Figure 3A). All samples reached a measurable RNA concentration by Qubit analysis. The RNA content of myotube derived EVs and the parental cells, before and after EPS, was analyzed using the GeneChip™ miR Arrays 4.0 in combination with Flash Tag™ Biotin RNA labelling revealing signal values ranging from 2 to 37000.

#### Distribution of non-coding RNA cargo in exosomes and MV from unstimulated myotubes

Based on an initial input of 2.5 ng EV RNA from unstimulated myotubes and filtration for signal values ≥ 5, several groups of non-coding RNA transcripts were identified, including miRNA (miR), stem-loop miRNA, CDBOX (U43), snoRNA, and 5.8 s rRNA (Figure 3B). The total number of EV miRs detected was approximately 400, where almost 170 out of these transcripts were shared between exosomes and MV (Figure 3C, Supplementary Table S5A). The small sized RNA fraction isolated from remaining myotubes contained approximately 360 miRs, where about half of this miR cargo was present in both exosomes and MV. An exosome profile of 30 distinct miRs was observed compared to MV and myotubes (Figure 3C, Supplementary Table S5B), whereas the miR pattern in MV strongly mirrored the miR pattern observed in the parental myotubes (correlation coefficient MV vs. MYO = 0.96).

#### Effect of EPS on miRNA cargo in exosomes and MV

**FIGURE 4:**
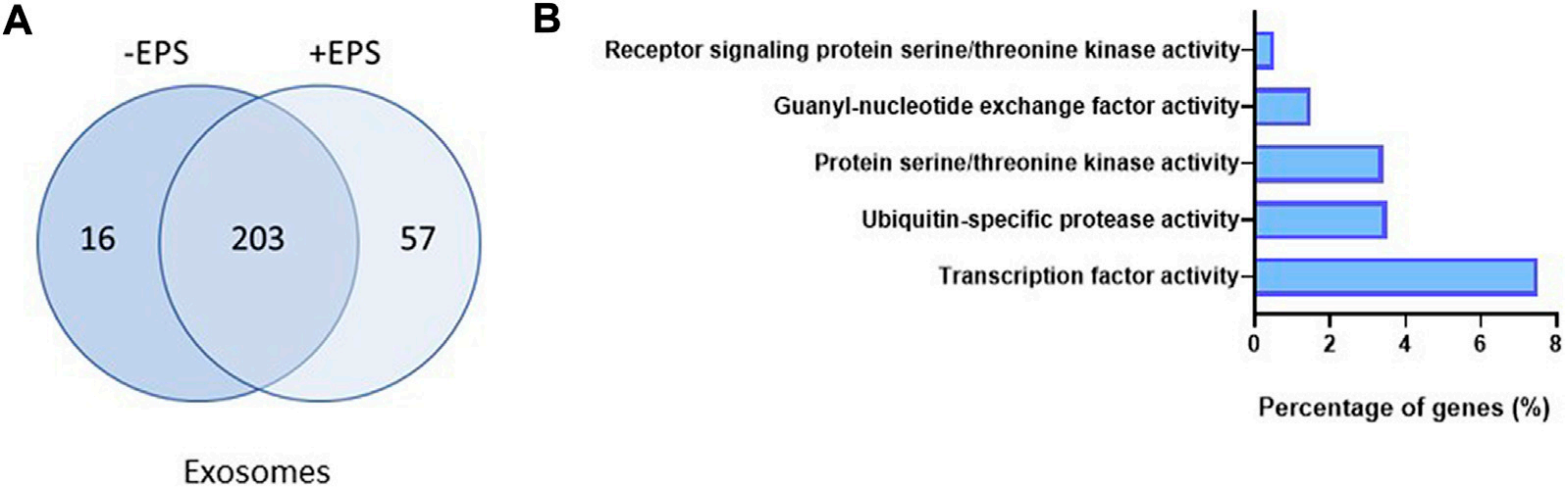
Exosome miRs affected by electrical pulse stimulation (EPS). The number and distribution of miRs present in exosomes before and after EPS **(A)**. Enrichment analysis of the miRs in exosomes after EPS (performed by FunRich) which shows the predicted molecular functions significantly associated (*p* < 0.05) with the miRs **(B)**.

Comparing the distribution of EV miR transcripts before and after EPS, disclosed that the exosomes changed the miR content more than the MV. The total number of miRs in exosomes increased from 219 to 260 (Figure 4A, Supplementary Table S5A,C), whereas the miR pattern in MV were almost unchanged. In exosomes, based on signal values ≥ 5 and fold change ≥1.5, the quantity of 15 specific miRs were significantly changed after EPS (Table 1), whereas the level of miRs in MV showed to be less affected than exosomes. A miR enrichment analysis (FunRich) revealed that the present collection of exosome miRs after EPS were significantly associated with molecular functions associated with cellular transcription, ubiquitin-specific protease activity, and serine/threonine kinase activity (Figure 4B). Analysis of myotube RNA concluded that 16 miRs were significantly increased after EPS, but none of them reached the threshold of ≥1.5-fold change (Supplementary Table S5A).

**TABLE 1:** Exosome miRs significantly changed by EPS.

Transcript Id	*p*-value	Fold change by EPS
miR-1233-5p	0.015	3.29
miR-320b	0.033	2.81
miR-4532	0.035	2.52
miR-92b-5p	0.026	2.49
miR-3141	0.025	2.35
miR-4459	0.026	2.26
miR-4649-5p	0.004	2.02
miR-4467	0.037	1.98
miR-4433b-3p	0.034	1.89
miR-1909-3p	0.038	1.78
miR-2277-5p	0.038	1.68
miR-6749-5p	0.008	1.65
miR-3188	0.036	1.60
miR-6805-5p	0.015	1.59
miR-6735-5p	0.032	1.52

Signal value ≥5.

### Proteomic analysis of exosome and MV cargo

#### Protein cargo of exosomes and MV from unstimulated myotubes

**FIGURE 5:**
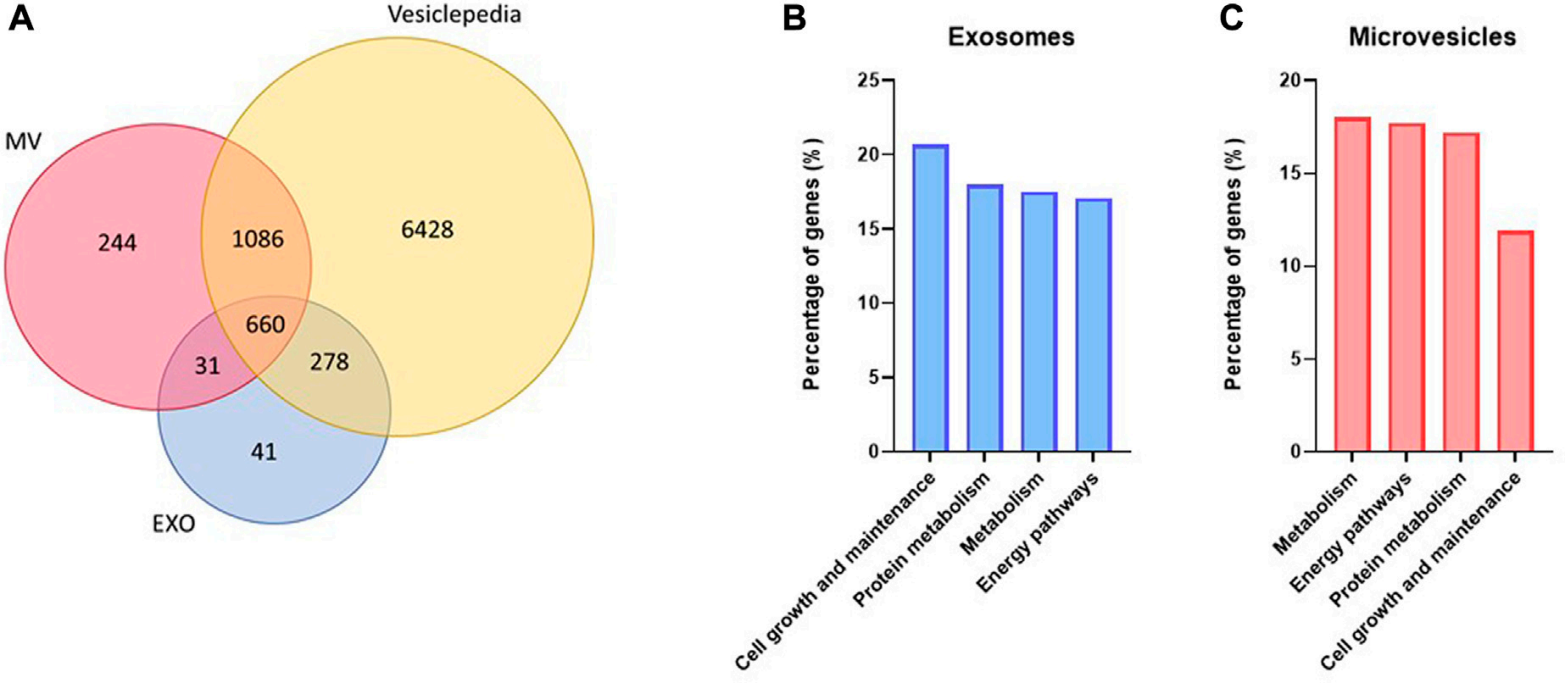
Protein content in unstimulated myotube derived extracellular vesicles. Extracellular vesicles were isolated from human myotube cell media after 24 h collection. The protein content of both exosomes (EXO) and microvesicles (MV) were detected by nanoLC-MS/MS and identified by MaxQuant against the Uniprot database. About 1000 proteins were detected in EXO and 2000 in MV, and most of these were found in Vesiclepedia **(A)**. FunRich was used to predict which biological processes the detected proteins were significantly (*p* < 0.01) associated with in exosomes **(B)** and MV **(C)**.

Quantitative, high-resolution mass spectrometry-based proteomics was used to identify and characterize the exosome and MV protein cargo. Approximately 1000 and 2000 proteins were identified from exosome and MV samples, respectively (Figure 5A, Supplementary Tables S6A, C). Comparison of our proteomic datasets with Vesiclepedia (by November 2022) showed that most of the proteins identified were known components of EVs (Figure 5A). Classification of the proteins based on ‘biological function’ (Figures 5B,C) showed that in exosomes, proteins related to cell growth and maintenance, protein metabolism, metabolism and energy pathways were the most abundant (Figure 5B). Proteins in MV were mostly related to metabolism, energy pathways, protein metabolism, and cell growth and maintenance (Figure 5C). Importantly, also proteins related to skeletal muscle development and contraction were observed in both exosomes and MV (Supplementary Tables S6A, C).

#### Effect of EPS on protein cargo of exosomes and MV

EPS significantly changed the protein cargo of both exosomes and MV. In exosomes, 48 proteins were detected in higher content and 25 in reduced content after EPS (Supplementary Table S6B), while in MV 65 proteins were elevated and 32 lowered by EPS (Supplementary Table S6D). The exosome proteins that were most affected by EPS (≥1.5-fold) are given in Table 2, and the MV proteins (≥1.5-fold) are given in Table 3. From these proteins, four were changed in both exosomes and MV, although not 1.5-fold in MV: Neutral alpha-glucosidase AB (GANAB), stress-70 protein mitochondrial (HSPA9), cytosolic non-specific dipeptidase (CNDP2) and ATP synthase subunit beta mitochondrial (ATP5B).

**TABLE 2:** The exosome proteins that were most (>1.5-fold) changed by EPS.

Protein	Name	*p*-value	Fold change
BAG2	BAG family molecular chaperone regulator 2	0.008	4.62
MEGF10	Multiple epidermal growth factor-like domains protein 10	0.031	4.43
GANAB	Neutral alpha-glucosidase AB	0.005	3.10
PSME2	Proteasome activator complex subunit 2	0.031	2.58
OGDH	2-oxoglutarate dehydrogenase, mitochondrial	0.014	2.41
AHNAK	Neuroblast differentiation-associated protein AHNAK	0.005	2.18
HSPA9	Stress-70 protein, mitochondrial	0.010	2.15
SYNPO2	Synaptopodin-2	0.046	2.13
ZYX	Zyxin	0.036	2.09
HNRNPK	Heterogeneous nuclear ribonucleoprotein K	0.014	2.02
WARS	Tryptophan--tRNA ligase, cytoplasmic; T1- TrpRS; T2-TrpRS	0.009	2.01
AARS	Alanine--tRNA ligase, cytoplasmic	0.030	2.00
TFRC	Transferrin receptor protein 1	0.045	1.97
CNDP2	Cytosolic non-specific dipeptidase	0.031	1.96
FAM129B	Niban-like protein 1	0.048	1.95
RNH1	Ribonuclease inhibitor	0.014	1.89
CAP1	Adenylyl cyclase-associated protein 1	0.009	1.87
MAP4	Microtubule-associated protein 4	0.002	1.73
RPS15A	40S ribosomal protein S15a	0.010	1.67
GSTO1	Glutathione S-transferase omega-1	0.048	1.66
SND1	Staphylococcal nuclease domain-containing protein 1	0.041	1.63
TPT1	Translationally-controlled tumor protein; TPT1-like protein	0.040	1.58
F13A1	Coagulation factor XIII A chain	0.003	1.57
ATP5B	ATP synthase subunit beta, mitochondrial	0.007	1.52
S100A7; S100A7A	Protein S100-A7; Protein S100-A7A	0.043	−2.49
VNN2	Pantetheine hydrolase VNN2	0.001	−1.74
ITGBL1	Integrin beta-like protein 1	0.010	−1.63
NCAM1	Neural cell adhesion molecule 1	0.006	−1.55
APOE	Apolipoprotein E	0.012	−1.53
BMP1	Bone morphogenetic protein 1	0.006	−1.51

**TABLE 3:** The MV proteins that were most (>1.5-fold) changed by EPS.

Protein	Name	*p*-value	Fold change
GNAQ	Guanine nucleotide-binding protein G(q) subunit alpha	0.042	2.90
NCLN	Nicalin	0.021	2.64
CYC1	Cytochrome c1, mitochondrial	0.009	1.98
RETSAT	All-trans-retinol 13,14-reductase	0.021	1.98
GOLGA3	Golgin subfamily A member 3	0.031	1.83
SUCLG1	Succinyl-CoA ligase [ADP/GDP-forming] subunit alpha, mitochondrial	0.048	1.74
RAP2C	Ras-related protein Rap-2c	0.034	1.60
DLAT	Dihydrolipoyllysine-residue acetyltransferase component of pyruvate dehydrogenase complex, mitochondrial	0.017	1.59
MOGS	Mannosyl-oligosaccharide glucosidase	0.010	1.53
TOMM70A	Mitochondrial import receptor subunit TOM70	0.014	1.51
MAMDC2	MAM domain-containing protein 2	0.008	−2.39
LGALS3BP	Galectin-3-binding protein	0.002	−2.06
MAP2K3	Dual specificity mitogen-activated protein kinase kinase 3	0.036	−2.06
SKP1	S-phase kinase-associated protein 1	0.003	−2.04
COL1A1	Collagen alpha-1(I) chain	0.010	−1.98
C1R	Complement C1r subcomponent; Complement C1r subcomponent heavy chain; Complement C1r subcomponent light chain	0.019	−1.97
COL1A2	Collagen alpha-2(I) chain	0.003	−1.91
C1S	Complement C1s subcomponent; Complement C1s subcomponent heavy chain; Complement C1s subcomponent light chain	0.010	−1.79
LAMB1	Laminin subunit beta-1	0.031	−1.74
LAMA2	Laminin subunit alpha-2	0.022	−1.59
LAMC1	Laminin subunit gamma-1	0.015	−1.57
VCAN	Versican core protein	0.010	−1.56
SRPX2	Sushi repeat-containing protein SRPX2	0.046	−1.53

**FIGURE 6:**
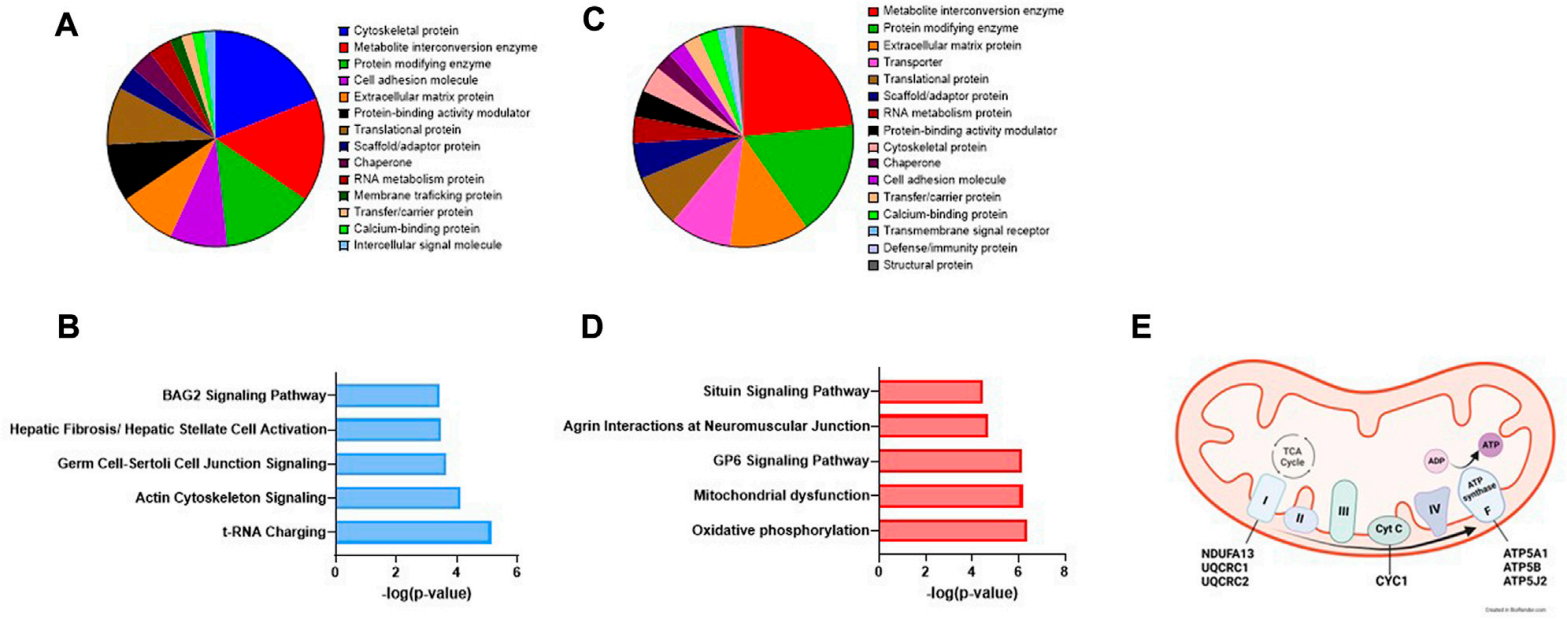
Panther classification and Ingenuity Pathway analysis (IPA) of extracellular vesicle proteins changed by EPS. Of the 73 proteins that were changed by EPS in exosomes, 60 were classified into protein classes by the Panther classification system **(A)**. The top predicted canonical pathways of proteins regulated by EPS in exosomes assessed by IPA **(B)**. Panther classification of 77 of the 95 proteins changed by EPS in MV **(C)**, the top canonical pathways of the proteins changed by EPS in MV **(D)**, and the proteins of the mitochondrial oxidative phosphorylation pathway activated by EPS in MV **(E)**.

To gain biological significance of the proteins changed by EPS, we next performed Panther classification and Ingenuity Pathway Analysis (IPA) for all the proteins that were significantly affected by EPS in exosomes and MV. About half of the proteins affected by EPS in exosomes belonged to the protein classes cytoskeletal proteins, metabolite interconversion enzymes, and protein modifying enzymes (Figure 6A), whereas the pathways predicted to be most affected were tRNA charging and actin cytoskeleton signaling (Figure 6B). In MVs the largest protein classes were metabolite interconversion enzymes, protein modifying enzymes, and extracellular matrix proteins (Figure 6C), while oxidative phosphorylation, mitochondrial dysfunction, and GP6 (platelet glycoprotein VI) signaling were predicted to be the pathways most affected (Figures 6D,E).

### Interactions of miRNAs and proteins in exosomes

Based on the innate capacity of miRs to bind specific mRNA targets and thereby facilitate translational regulation, a search for the presence of affected target proteins in the exosome material was performed. The 15 miRs with significantly changed levels after EPS (Table 1) were analyzed against the 73 proteins observed with significantly different levels after EPS (Table 2; Supplementary Table S6B) using IPA. A total of 31 target proteins of the selected miRs were identified (Table 4). Out of all proteins detected with miR match, 11 proteins showed a lowered quantity with fold changes from −1.1 to −2.5, whereas 20 of them were elevated with a fold change range from 1.5-3.1. Most of the miRs matched with more than one target protein, and several target proteins matched with more than one miR. Of all the miR target protein pairs observed, nine were predicted with a high confidence level, whereas the rest were predicted at moderate confidence levels (Table 4).

**TABLE 4:** Matches between significantly changed miRs and target proteins in exosomes after EPS.

microRNA IDs	Confidence	Gene names	Protein names	Fold change
miR-1233-5p	High (predicted)	GANAB	Neutral alpha-glucosidase AB	3.10
miR-1233-5p	High (predicted)	S100A7	Protein S100-A7	−2.49
miR-1233-5p	Moderate (predicted)	CLSTN1	Calsyntenin-1	−1.14
miR-1233-5p	Moderate (predicted)	COL6A1	Collagen alpha-1(VI) chain	−1.14
miR-1233-5p	Moderate (predicted)	VCL	Vinculin	1.26
miR-1233-5p	Moderate (predicted)	PVR	PVR Cell Adhesion Molecule	−1.45
miR-1233-5p	Moderate (predicted)	SYNPO2	Synaptopodin-2	2.13
miR-320b	High (predicted)	TFRC	Transferrin receptor protein 1	1.97
miR-320b	Moderate (predicted)	WARS	Tryptophan-tRNA ligase	2.01
miR-4532	Moderate (predicted)	CLSTN1	Calsyntenin-1	−1.14
miR-4532	Moderate (predicted)	PLEC	Plectin	1.26
miR-4532	Moderate (predicted)	EEF2	Eukaryoticelongation factor2	1.30
miR-4532	Moderate (predicted)	SEMA7A	Semaphorin 7A	−1.41
miR-4532	Moderate (predicted)	MAP4	Microtubule-associated protein 4	1.73
miR-4532	Moderate (predicted)	RPS15A	40S ribosomal protein S15a	1.67
miR-92b-5p	Moderate (predicted)	CLSTN1	Calsyntenin-1	−1.14
miR-92b-5p	Moderate (predicted)	VCL	Vinculin	1.26
miR-92b-5p	Moderate (predicted)	SND1	Staphylococcal nuclease domain-containing protein 1	1.63
miR-4649-5p	High (predicted)	ZYX	Zyxin	2.09
miR-4433b-3p	High (predicted)	TFRC	Transferrin receptor protein 1	1.97
miR-4433b-3p	Moderate (predicted)	COL6A1	Collagen alpha-1(VI) chain	−1.14
miR-4433b-3p	Moderate (predicted)	CNDP2	Cytosolic non-specific dipeptidase	1.96
miR-1909-3p	High (predicted)	MAP1A	Microtubule AssociatedProtein1A	1.28
miR-1909-3p	High (predicted)	RNH1	Ribonuclease inhibitor	1.89
miR-1909-3p	Moderate (predicted)	SERPING1	Plasma protease C1 inhibitor	−1.22
miR-1909-3p	Moderate (predicted)	CSF1	Macrophage colony-stimulating factor 1	−1.22
miR-1909-3p	Moderate (predicted)	DES	Desmin	1.28
miR-1909-3p	Moderate (predicted)	GSN	Gelsolin	1.31
miR-1909-3p	Moderate (predicted)	BMP1	Bone morphogenetic protein 1	−1.52
miR-1909-3p	Moderate (predicted)	FAM129B	Niban-like protein 1	1.95
miR-1909-3p	Moderate (predicted)	TFRC	Transferrin receptor protein 1	1.97
miR-2277-5p	High (predicted)	APOE	Apolipoprotein E	−1.54
miR-2277-5p	Moderate (predicted)	SDF4	45 kDa calcium-binding protein/Stromal cell-derived factor 4	−1.25
miR-2277-5p	Moderate (predicted)	ZYX	Zyxin	2.09
miR-6749-5p	Moderate (predicted)	CLSTN1	Calsyntenin-1	−1.14
miR-6749-5P	Moderate (predicted)	PVR	PVR Cell Adhesion Molecule	−1.45
miR-6805-5p	High (predicted)	MAP4	Microtubule-associated protein 4	1.73
miR-6805-5p	Moderate (predicted)	COL6A1	Collagen alpha-1(VI) chain	−1.14
miR-6805-5p	Moderate (predicted)	CSF1	Macrophage colony-stimulating factor 1	−1.22
miR-6805-5p	Moderate (predicted)	WARS	Tryptophan--tRNA ligase	2.01
miR-6805-5p	Moderate (predicted)	GANAB	Neutral alpha-glucosidase AB	3.10
miR-6735-5p	Moderate (predicted)	PLOD3	Procollagen glycosyltransferase	1.13
miR-6735-5p	Moderate (predicted)	CSF1	Macrophage colony-stimulating factor 1	−1.22
miR-6735-5p	Moderate (predicted)	VCL	Vinculin	1.26
miR-6735-5p	Moderate (predicted)	BMP1	Bone morphogenetic protein 1	−1.52
miR-6735-5p	Moderate (predicted)	ITGBL1	Integrin beta-like protein 1	−1.64
miR-6735-5p	Moderate (predicted)	RPS15A	40S ribosomal protein S15a	1.67
miR-6735-5p	Moderate (predicted)	MAP4	Microtubule-associated protein 4	1.73
miR-6735-5p	Moderate (predicted)	AARS	Alanine--tRNA ligase, cytoplasmic	2.00

## Discussion

T2D has a strong genetic predisposition, which may be unmasked by lifestyle factors such as obesity and lack of exercise. Thus, T2D is potentially preventable in a substantial proportion of people. Initially, T2D may be controlled through a healthy diet, weight loss, and physical activity. Exercise has been proven to be one of the most efficient drivers of a better health in human studies (Arem et al., 2015; Luan et al., 2019; Chow et al., 2022), and lifestyle disorders like obesity, T2D, and cardiovascular disease have been shown to be prevented and treated by regular physical activity. The mechanisms of how exercise contribute to the positive physiological effect is still not fully understood, but it is well documented that skeletal muscles are involved in cellular communication through a myriad of secreted components.

To our knowledge, this is the first study to unravel the effect of electrical pulse stimulation (EPS) on the total miR and protein cargo components of extracellular vesicles (EVs) secreted from human myotubes. We have used myotubes cultured from biopsy samples taken during bariatric surgery of morbidly obese, female donors with T2D. By exploring the total miR and protein content of myotube derived EVs, we show that EPS significantly changed the level of 15 miRs in exosomes, 73 proteins in exosomes and 97 proteins in MVs. From these proteins, four were changed in both exosomes and MVs (GANAB, HSPA9, CNDP2, ATP5B). IPA revealed that the 15 miRs may target 31 of the EPS-modified proteins present in exosomes, and the highly predicted targets S100A7 (miR-1233-5p) and APOE (miR-2277-5p) were both found at reduced levels. Of the four proteins changed by EPS in both exosomes and MVs, GANAB and CNDP2 were also targets of EPS-changed miRs (miR-1233-5p and miR-6805-5p, and miR-4433-3p, respectively). Based on the recent observation of Lac-Phe as an appetite regulator generated by exercise (Li et al., 2022a), the present finding of increased content of CNDP2 in EVs from skeletal muscle in response to EPS is particularly interesting. Several of the other miRs we found changed by EPS, such as miR-92b-5p and miR-320b, might be promising signaling factors of exercise.

Although EVs have been isolated from unstimulated human skeletal muscle cells before (Le Bihan et al., 2012; Le Gall et al., 2020), and also after EPS (Vechetti et al., 2021), we are the first to do untargeted search for the complete protein and miR content of EVs after EPS. Le Bihan et al. showed, using a transcriptomic and a proteomic approach, that human skeletal muscle cells secrete EVs during differentiation of myoblasts to myotubes (Le Bihan et al., 2012). In accordance with Le Bihan et al., we found distinct RNA and protein cargo in exosomes and MV derived from unstimulated skeletal muscle cells. In our study, the miR profiles of human myotube derived exosomes *versus* MV and myotubes were clearly different and may represent miR patterns specific for the diseased patient with morbid obesity and T2D.

According to the microarray results from unstimulated cells, a total of approximately 400 miRs were identified in the EVs. Most of them were present in both exosomes and MV, as well as in myotubes, and many at high signal values, such as miR-4497, miR-4787, miR-3665, miR-3960, miR-6087, miR-6089, miR-6090, and miR-7704 (Supplementary Table S5A), making a pattern where highly enriched miRs in the parental myotubes were mirrored in the derived EVs. The proof of concept that “EVs are a snapshot of their parental cell” was substantiated. Other miRs did however show a divergent pattern. Whereas all the MV miRs were shared with either exosomes or myotubes, 30 distinct miRs were detected in exosomes only (Supplementary Table S5B), thereby generating a specific exosome miR profile. Interestingly, miR-224 was present with the highest signal level of all in the unique exosome list. As far as we know, miR-224 has not been associated with T2D or obesity, but it may be a lifestyle disease marker in exosomes from skeletal muscles. The highest predicted target proteins of miR-224 are CDK8 and PYROXD1, known as an active regulator of gene transcription and a mitochondrial oxidoreductase in the electron transport chain, respectively. Sullivan et al. analyzed miRs from small EVs derived from skeletal muscle biopsies generated from obese and lean, but otherwise healthy, men and women (Sullivan et al., 2022). RNA sequencing revealed that 22 of the miRs appeared in different amounts in the two groups. Comparing those to the 219 miRs detected in our unstimulated exosomes (Supplementary Table S5C), several miRs from the obese group were common (miR-let-7f-5p, miR-337-3p, miR-3613, miR-548-3p, miR-7641 and miR-7977). In addition, 10 out of Sullivan´s 22 reported miRs were present in our myotubes, and one of these, miR-7977, were present also in exosomes and MV. Previously, miR-7977 has been associated with hyperinsulinemia and impaired glucose tolerance induced renal injury (Gao et al., 2020), indicating an interesting role for this miR in the actual patient group.

Until recently, the majority of the skeletal muscle derived EV studies have focused on myomiR regulation. MyomiRs are thought to be of the most abundant miRs in myotubes, as well as in their derived EVs. Our results confirmed that some of the myomiRs, such as miR-1, miR-133a, and miR-486 were present in myotubes, but not in the myotube derived EVs. MyomiR-206 was found in both myotubes, exosomes, and MV (Supplementary Table S5A), but at a very high level only in exosomes. Moreover, Massart et al. reported that also the miR-let-7 family, consisting of miR-let-7a, -7b, -7c and -7f, but also miR-23a and miR-23b, are present at high levels in biopsies from unstimulated skeletal muscle (Massart et al., 2021). In the present study, both exosomes, MV, and myotubes contained the entire series of both the miR-let-7 and miR-23, but at variable levels, some highly abundant, whereas other at lower level. Skeletal muscle cells are highly adaptable, and the myomiRs have been shown to be important for rapid activation and differentiation of the muscle cells after different kind of stimuli. Cellular regulation of the myomiR quantity may therefore be of functional importance for the muscle itself, and selective loading of myomiRs into EVs may be a way of intercellular communication. Uptake of EVs loaded with myomiRs may directly initiate translational downregulation and reprogramming of the recipient cell after binding of myomiRs to their target mRNAs.

The miR cargo of MV was almost a mirror image of the miRs in myotubes (correlation coefficient 0.96), implying that enriched cellular miRs may be easily loaded up during the outward budding process of MV. According to Garcia-Martin et al., the balance between release or cellular retention of exosomes and their miR cargo seems to be more carefully regulated than MV (Garcia-Martin et al., 2022). Data indicate that there is an active, complex, and integrated system, involving multiple sequence motifs in the miRs. These may contribute to sorting of the miRs into exosomes or small EVs (EXOmotifs) or cellular retention (CELLmotifs) in a cell type specific way (Garcia-Martin et al., 2022). Interestingly, the skeletal muscle cell line C2C12 was used as an example of the principle, presenting eight different overrepresented EXOmotifs. Several of the 15 significantly elevated miRs in our exosomes carried one or more of the tetra or extended sequences (GGAG, AGGG, UGUG, GGGAG, GAGGC, UGGGAG, GAGGGC) reported to be present in the C2C12 cell line (Garcia-Martin et al., 2022). Increased miR delivery mediated by EXOmotifs will lead to an enhanced regulation potential of their target genes.

Most of the proteins we observed in EVs from unstimulated myotubes were common for the two vesicle types, but distinct proteins of exosomes and MV were identified (Figure 2 and Supplementary Tables S6A, C). The difference between proteins, as well as miRs, in exosomes and MV can be ascribed to differences in biogenesis of exosomes and MV and protein sorting mechanisms involved (Lischnig et al., 2022). Moreover, it may also confirm well separated EV fractions. The biological processes associated with the exosome and MV proteins were however overlapping, where cell growth and maintenance dominated in exosomes and metabolism in MV. The predicted pathways we found significantly associated were in accordance with the molecular and cellular functions described by Le Bihan et al. (Le Bihan et al., 2012), despite different sample material. Most of the proteins found in human skeletal muscle cell derived EVs were previously described in human Vesiclepedia, although no other studies on human myotubes or skeletal muscle were found in Vesiclepedia.

In the present study, EPS had no effect on EV size and number, but clearly changed the EV content of both miRs and proteins. Plasma EV have been thought to increase in number by exercise (Estebanez et al., 2021), but the source of these might not be skeletal muscle alone since *in vitro* treatment of skeletal muscle cells with EPS did not seem to increase EV number (Vechetti et al., 2021). However, uncertainty in particle isolation methods and counting techniques must be beard in mind, as well as great variability in exercise protocols and timing of EV sampling. In our study, EPS significantly changed the level of 15 specific miRs in exosomes, but none in MVs. In myotubes, 16 miRs were significantly affected by EPS (Supplementary Table S5A), and only one of the miRs, miR-92b-5p, that increased in myotubes were also changed by EPS in exosomes. To our knowledge, the whole miR content of human myotube derived EVs in response to EPS has not been thoroughly explored before. However, a targeted search for miR-1, known to have a functional role in developing skeletal muscle precursor cells (Khan et al., 2022), showed an increase in both EPS stimulated human myotube-derived EVs and in plasma EVs after 30 min resistance exercise (Vechetti et al., 2021). In the present study, miR-1 was found in myotubes, but was neither changed by EPS, nor found in exosomes or MV. This could be due to differences in EV separation techniques and another EPS protocol. Recently, EVs were isolated from human skeletal muscle biopsies after exercise training (Massart et al., 2021; Sullivan et al., 2022). None of the miRs found at different level after 1 week exercise training in lean and obese sedentary individuals (Sullivan et al., 2022) or after a 14-day aerobic exercise training intervention of healthy, sedentary men (Massart et al., 2021) were changed in our *in vitro* exercise model. Nevertheless, quite a few of the 28 mentioned miRs found in obese after exercise in Sullivan’s study (Sullivan et al., 2022) were present in our exosomes (7), MV (4), and myotubes (11), where miR-23a, miR-3960, miR-4488 and miR-4497 were detected with the highest signal values of all our miRs in MV and myotubes. In the mentioned *in vivo* exercise intervention studies (Massart et al., 2021; Sullivan et al., 2022), EVs were collected directly from skeletal muscle biopsy samples taken 12–14 h and 16 h post-exercise, respectively. The cellular source of EVs from biopsy samples could be heterogenous, and as documented by Massart et al., the miR expression in cultured skeletal muscle cells differed from the tissue samples (Massart et al., 2021). The human myotube cultures used in the present study are free of other contaminating cells, and consequently, the EVs collected from the cell culture media must be muscle derived. Even though the origin of EVs is unquestionable, the myotubes might not behave as *in vivo* skeletal muscle, particularly not in response to exercise/EPS. We have previously argued that the EPS model mimic exercise quite well, at least when it comes to metabolic effects (Nikolic et al., 2017). Recently, a comparative analysis of exercise signature genes in skeletal muscles after *in vivo* exercise and *in vitro* EPS also confirmed that the EPS model mimicked *in vivo* exercise at the transcriptome level (Lee et al., 2022). Altogether, the EPS model of human myotubes appears to be useful and relevant. Further, it must be emphasized that our myotubes were from morbidly obese and T2D donors, and we know from previous studies that myotubes from obese tend to respond differently to EPS than myotubes from lean (Feng et al., 2015). We cannot exclude that other miRs and proteins would be changed by EPS in myotubes from lean and glucose tolerant individuals. However, we believe the present results represent true EPS effects and are valid for the actual patient group.

Of the miRs that we found significantly increased by EPS, particularly miR-92b-5p, miR-320b, and miR-1233-5p were interesting. Reduced level of mir-320b has been suggested as a serum marker for both type 2 diabetes (Yan et al., 2016) and carotid atherosclerosis (Zhang et al., 2016). The finding that EPS increases miR-320b might therefore be noteworthy. Several studies on exercise and plasma EVs have been reported (Estebanez et al., 2021; Doncheva et al., 2022). The review by Estebanez et al. summarized results of released exosome miRs in plasma after four different exercise protocols (Estebanez et al., 2021). Common for these studies were detection of mainly well-known myomiRs at varying concentrations, and some examples of the most highly abundant miRs, like miR-23a and -b and miR-29b. Beyond these findings, the results are quite mixed with separate candidate miRs popping up as expected, as training profiles, isolation methods, and miRNA analyses vary between the studies. In addition, plasma is a complex body fluid to analyze for signature miRs because all body cells excrete their EVs into the circulation, creating a big heterogeneous EV pool. None of the plasma miRs described by Estebanez et al. matched with our 15 significantly changed miRs in exosomes from patient derived myotubes (Estebanez et al., 2021). MiR-92b-5p might also be interesting, since it recently showed higher expression in healthy active individuals than in sedentary individuals (Streese et al., 2022), indicating a role in physical activity/exercise. MiR-1233-5p was the miR most changed by EPS in our study, and the highly predicted target protein S100A7 was the protein most reduced after EPS. S100A7 is a member of a Ca^2+^-binding protein family that has been shown to interact with miRNAs (Ghafouri-Fard et al., 2022), and is over-expressed in psoriatic lesions, probably involved in cell proliferation, differentiation, and inflammation (D'Amico et al., 2016). To our knowledge S100A7 has not been associated with exercise before, but our results indicate that miR-1233-5p might repress S100A7, and thereby, we can speculate about a role in the anti-inflammatory effect of exercise.

Both experimental and computer analysis indicate that most protein coding genes, through their mRNAs, are regulated by one or more miRs. Prediction of functional miR targets by the target prediction database (miRDB) (Liu and Wang, 2019), revealed more than 4000 theoretical targets altogether for 13 of the 15 miRs that were changed by EPS (Tab. 1). Available prediction algorithms still have suboptimal performance often leading to false predictions (Liu and Wang, 2019), and our target search of the miRs resulted in 31 protein matches. However, worth of notice, this target search was performed only with miRs and proteins collected in the same exosome cargo, which is probably not ideal for assessment of miR specificity. Such an analysis may nevertheless give valuable information on the condition of the mother cell, but even more important, contribute to an increased insight into the various downstream pathways of EPS-changed miRs. The pathway analysis of EPS changed miRs in exosomes (Figure 4B) suggests strong involvement in several cell signaling functions.

The effect of EPS was evident on protein content of both exosomes and MV. In exosomes many cytoskeletal proteins were affected, as well as metabolite interconversion enzymes, and protein modifying enzymes, while the biological processes involved were tRNA charging and actin cytoskeletal signaling. This makes sense considering cytoskeletal rearrangement associated with contraction and the growth promoting effect of exercise. In MV, the main protein classes were enzymes involved in metabolite interconversion and protein modifications, and the pathways involved were oxidative phosphorylation, mitochondrial dysfunction, and GP6 signaling pathway. We and others have previously shown that EPS increases both glucose and fatty acid oxidation in human myotubes (Nikolic et al., 2012; Feng et al., 2015) and increases mitochondrial biogenesis (Fiorenza et al., 2019). Thus, the protein content of MVs from EPS treated myotubes reflects the state of their cell of origin.

Of the proteins detected in EVs after EPS, new exercise factors might be identified. The four proteins that were changed in both exosomes and MV, GANAB, HSPA9, CNDP2, and ATP5B, stand out as obvious candidates. Two of these were in addition targets of the EPS regulated miRs miR-1233-5p (GANAB), miR-6805-5p (GANAB), and miR-4433-3p (CNDP2). However, the protein GANAB and CNDP2 were not repressed by the respective miRs in our EVs, suggesting regulatory functions on other distant targets by these miRs. GANAB (glucosidase II alpha subunit) plays a role in protein folding and quality control by cleaving glucose residues from immature glycoproteins in the endoplasmic reticulum (NIH, National library of Medicine). GANAB has been found as one of six genetic loci associated with body shape (Ried et al., 2016). The role of GANAB in exercise is not known. HSPA9 (mitochondrial 70 kDa heat shock protein, mortalin) is a chaperon protein which plays a role in mitochondrial iron-sulfur cluster biogenesis (UniProt). To the best of our knowledge, there are no previously known associations between HSPA9 and exercise or physical activity. ATP5B (ATP synthase subunit beta) is a mitochondrial protein, and increased expression by EPS is not surprising. Similar EPS treatment of myotubes is known to improve mitochondrial function (Nikolic et al., 2012; Feng et al., 2015), increase mitochondrial content (Nikolic et al., 2012), and OXPHOS protein expression (Feng et al., 2015). The finding of CNDP2 (cytosolic non-specific dipeptidase) in both exosomes and MV, and the presence of miR-4433b-3p, which targets CNDP2, is, however, very interesting. CNDP2 is an enzyme that catalyzes the synthesis of Lac-Phe (an amidated conjugate of lactate and phenylalanine). Lac-Phe has been found increased in plasma of humans after a bout of strenuous exercise (Jansen et al., 2015), and recently, it was shown to be increased in plasma of two independent animal models of exercise (mice and racehorses) and in humans doing both endurance, sprint, and resistance training (Li et al., 2022a). What is even more intriguing was that the same metabolite Lac-Phe when injected intraperitoneally, suppressed food intake and reduced body weight, as well as improved glucose homeostasis in obese mice (Li et al., 2022a). The CNDP2 enzyme is known as an intracellular enzyme with widespread expression. In the study by Li et al. (Li et al., 2022a), macrophages were suggested as the main source of Lac-Phe. However, we show here that CNDP2 is present in extracellular vesicles from EPS-treated myotubes, implying that contracting skeletal muscles could be a source of plasma CNDP2 and Lac-Phe production. Knowing that EPS also can increase the cell medium lactate concentration (Nikolic et al., 2012), the conditions for making Lac-Phe are arranged. MiR-4433b-3p which also is increased in exosomes from EPS-myotubes might play a regulatory role in Lac-Phe synthesis.

One specific single nucleotide variant (SNV) in CNDP2 has been found associated with obesity risk (Yamakawa-Kobayashi et al., 2017), whereas other SNVs appear more frequently in athletes (Guilherme and Lancha, 2017), implying a central role in metabolic regulation. The myotubes in the present study were established from obese subjects with established T2D, and it might be that CNDP2 plays a special role in EVs from obese. However, we have neither genotype nor enzyme activity information on CNDP2 in any of the donors in the present study. The presence of CNDP2 in EVs, as well as other proteins, is dependent on the origin and state of the parental cells. The amount of CNDP2 was increased in both exosomes (almost 2-fold) and MV (1.3-fold) after EPS, and the spreading of this enzyme though skeletal muscle derived EVs might contribute to systemic Lac-Phe production, better appetite regulation and weight controlling effects of exercise at least in obese. Li et al. also observed that Lac-Phe had no suppressive effect on food intake in lean mice, indicating a unique role in obesity (Li et al., 2022a).

Regular physical activity is a powerful regulator of metabolic health, and it is well known to improve glucose tolerance and insulin sensitivity in individuals with obesity and type 2 diabetes. The role of skeletal muscle derived EVs as communicating factors of systemic beneficial effects of exercise is, however, difficult to study *in vivo*. As discussed above, EPS-treatment of myotubes in culture is a model of exercise or physical activity with its strengths and limitations (Nikolic et al., 2017). However, an advantage of the present study is the methodological reductionism, which has been highly requested by the metabolic research community (Akbar et al., 2019). The practically pure cell system eliminates the uncertainty of EV origin. The effect of intervention (EPS) is easily measured in such cell cultures, but one must bear in mind that EPS might not fully explain exercise effects. Anyway, a complete overview of proteins and miRs changed by EPS, as presented in this study, will generate valuable results, and reveal new candidate signaling factors to be studied *in vivo*. Today, there are no consensus on cell specific EV markers, thus making it difficult to separately study EVs from skeletal muscles in plasma. Some of the miRs or proteins of EVs that we have revealed can possibly be used as biomarkers of skeletal muscle derived EVs. In addition, the myotubes in our study were from female donors with severe obesity and established type 2 diabetes which represent a highly relevant population. Nevertheless, the signaling function of EVs from EPS-treated myotubes must be verified. Uptake into relevant target cells and metabolic organs and improvement of metabolic functions, such as appetite regulation, must be shown.

**FIGURE 7:**
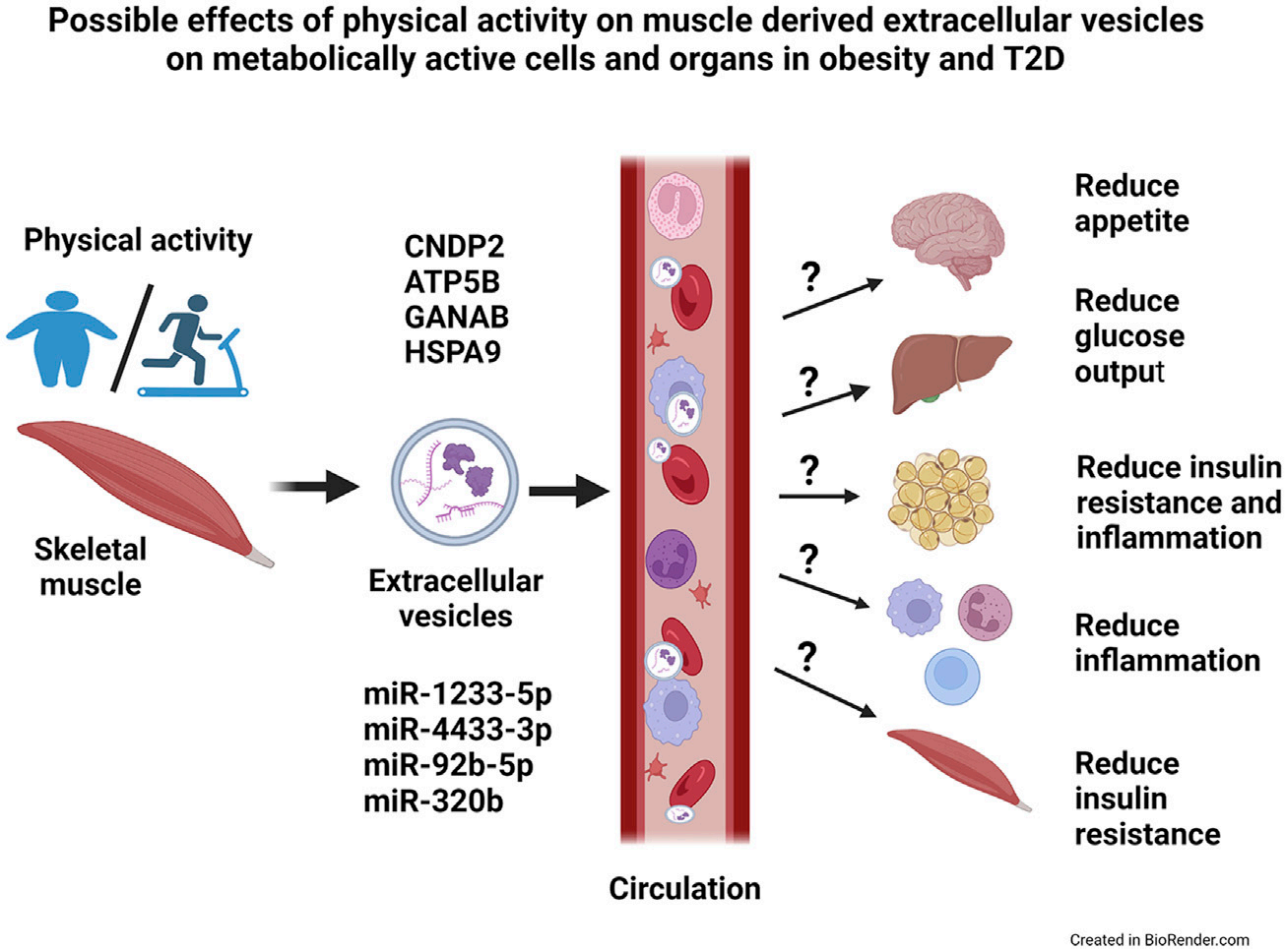
Possible effects of physical activity on skeletal muscle-derived extracellular vesicles on metabolically active cells and organs in obesity and type 2 diabetes (T2D). Skeletal muscles release extracellular vesicles (EVs) in response to exercise, and most likely will these EVs circulate and reach target organs. The EVs contain bioactive molecules, both microRNAs (miRs) and proteins, that might have a regulatory role on the metabolically active target cells and organs. Possibly, will EVs contribute to the health effects of exercise by reducing appetite, improve insulin sensitivity and reduce the low-grade inflammation in obesity and T2D. CNDP2 = cytosolic non-specific dipeptidase, ATP5B = ATP synthase subunit beta mitochondrial, GANAB = alpha-glucosidase AB, HSPA9 = stress-70 protein mitochondrial.

The aim of the present study was to get a broader picture of the global microRNA (miR) and protein content of extracellular vesicles (EVs) derived from human myotubes from morbidly obese patients with T2D, and study how this was changed by an *in vitro* exercise intervention (EPS). We used a descriptive approach and performed proteomic and transcriptomic analysis of the content of exosomes and MV. We found that these human myotubes secrete both exosomes and MV. EPS treatment of the myotubes, clearly changed the protein content of both exosomes and MV, whereas the miR content was changed only in exosomes. Size and number of EVs were not changed by EPS. Among the EPS-changed proteins and miRs, several promising signaling factors were identified (Figure 7). Of particular interest was CNDP2, an enzyme that can generate the appetite regulatory metabolite Lac-Phe. Among the regulated miRs, miR-92b-5p, miR-320b, miR-4433b-3p, and miR-1233-5p might also mediate signaling functions in target organs. Thus, skeletal muscles can produce EVs and change the EV content in response to EPS, and we suggest that skeletal muscle derived EVs can contribute to circulatory EVs and mediate beneficial effects of exercise in metabolically active organs (Figure 7). However, regulatory effects of these EVs must be experimentally verified.

## Data Availability

The mass spectrometry proteomics data have been deposited to the ProteomeXchange Consortium via the PRIDE [1] partner repository with the dataset identifier PXD040935". The microarray transcriptomics data has been deposited in NCBI's Gene Expression Omnibus (Edgar et al., 2002; Barrett et al., 2013) and are accessible through GEO Series accession number GSE227939. (https://www.ncbi.nlm.nih.gov/geo/query/acc.cgi?acc=GSE227939).
